# S-GRAS score and the complementary prognostic value of neutrophil-to-lymphocyte ratio in adrenocortical carcinoma: evidence for a synergistic interaction

**DOI:** 10.3389/fendo.2026.1733138

**Published:** 2026-06-08

**Authors:** Erik Bényei, Gergely Huszty, András Laki, Zsuzsanna Jakab, Gergely Kiss, Attila Kristóf Kovács, Katalin Borka, Andrea Uhlyarik, Katalin Eitler, Peter Igaz, Judit Tőke, Miklós Tóth

**Affiliations:** 1 Department of Internal Medicine and Oncology, Faculty of Medicine, Semmelweis University, Budapest, Hungary; 2 Department of Surgery, Transplantation and Gastroenterology, Faculty of Medicine, Semmelweis University, Budapest, Hungary; 3 Medical Imaging Centre, Faculty of Medicine, Semmelweis University, Budapest, Hungary; 4 Department of Pathology, Forensic and Insurance Medicine, Faculty of Medicine, Semmelweis University, Budapest, Hungary; 5 Department of Endocrinology, Faculty of Medicine, Semmelweis University, Budapest, Hungary

**Keywords:** adrenocortical cancer, inflammation-based score, neutrophil-to-lymphocyte ratio, platelet-to-lymphocyte ratio, prognostic model, S-GRAS score

## Abstract

**Introduction:**

This study aimed to assess the prognostic values of the S-GRAS model and two of the most frequently used inflammation-based scores – neutrophil-to-lymphocyte ratio (NLR) and platelet-to-lymphocyte ratio (PLR) – and to characterise the potential interaction between the two measures.

**Patients and methods:**

In this single-centre, retrospective, observational cohort analysis, 67 adult patients with histologically confirmed adrenocortical carcinoma (ACC) were analysed. The S-GRAS score and the inflammation-based markers were evaluated using Kaplan-Meier survival analysis with log-rank tests, univariate and multivariate Cox proportional hazards regressions. Discriminative ability was assessed using Harrell’s C-index, later compared with likelihood ratio tests. An interaction analysis was conducted by constructing a multivariate regression with mean-centred variables and their interaction term.

**Results:**

External validation on the S-GRAS scoring system demonstrated the superior discriminative ability of the model (C-index=0.765) compared to its constituents. Our sub-analysis on 42 patients identified NLR as a significant predictor of mortality (HR = 3.1; p=0.008), whilst PLR failed to reach statistical significance. Our interaction analysis revealed a significant synergistic interaction between S-GRAS and NLR (HR = 1.177, p=0.009). While both S-GRAS (C-index=0.803) and NLR (C-index=0.722) was found to be a significant predictor of survival, their joint application demonstrated superior prognostic discrimination (C-index=0.822) over their individual use (p=0.006 and p<0.001).

**Conclusions:**

NLR provides independent and complementary predictive value to the well-established S-GRAS score, and their combination offers superior discrimination over each marker alone. A synergistic interaction between the two metrics is the most prominent in high-risk patient groups. External validation confirms the prognostic robustness of the S-GRAS score.

## 1 Introduction

Adrenocortical cancer (ACC) is a rare malignant tumour with an incidence of 0.7 to 2 cases per million per year (1, 2). Although ACC has heterogeneous biological behaviour, it generally carries a poor prognosis (3–7). Several studies have attempted to identify associations between clinicopathological or laboratory features of ACC and overall survival (OS) or disease-free survival (DFS) (8–10). Understanding these associations could provide a basis for more personalised treatment approaches (10–12).

Recently, two scoring systems have been proposed incorporating individual prognostic factors significantly affecting survival (13–16). Libé et al. initially developed the GRAS score system for patients with advanced ENSAT stage III-IV disease (13). The components of the GRAS include grade (G), resection status (R), age (A), and tumour- or hormone-related symptoms (S). The scoring system was later modified to include adjustments applicable to all patients with ACC (17). The S-GRAS classification includes the ENSAT stage (S) and applies the Ki-67 index for grading. These scoring systems enhance prognostication by weighing and combining individual prognostic factors, thereby improving risk stratification. Based on the S-GRAS score, a management strategy has been proposed regarding postoperative adjuvant treatment, with mitotane alone or combined with cytotoxic drugs (18). The prognostic value of this system has been demonstrated in several large, multicentre studies (18–20), and a study featuring a patient cohort of a single centre (21). A summary of the clinicopathological characteristics of each patient cohort from previous studies is composed in **Supplementary Table 1**.

In recent years, increasing attention has been directed towards the applicability of various inflammation-based scores in clinical oncology research (22). Two frequently cited parameters of tumour-induced systemic inflammatory response are the neutrophil-to-lymphocyte ratio (NLR) and the platelet-to-lymphocyte ratio (PLR). A comprehensive review has been published on their prognostic value in endocrine tumours, suggesting that these markers may be useful in identifying aggressive disease courses and poor prognostic outcomes (23). Prognostic studies conducted on ACC patient cohorts have repeatedly identified an association between the values of these indeces and overall survival (24–27). A definitive methodology has yet to be established for evaluating inflammation-based scores in patients with ACC. Limited data are available regarding the comparison of the NLR and PLR with conventional prognostic factors, and, to the best of our knowledge, the relationship between inflammation-based, and the S-GRAS score has not yet been investigated.

The primary objective of this study was to evaluate the prognostic performance of the S-GRAS and inflammation-based scores, and to explore potential interactions between these measures. Specifically, we sought to determine whether these models represent complementary or overlapping predictive value, thereby clarifying whether their combined use offers added information over either approach applied alone. As a secondary aim, we validated the S-GRAS scoring system in an expanded cohort, representing the most extensive single-centre study to date.

## 2 Patients and methods

### 2.1 Patients and data collection

This single-centre, retrospective study included adult patients with histologically confirmed adrenocortical carcinoma treated at our centre between 2000 and 2023. This cohort partially overlaps with that published in 2022 (28) but have not been involved in any of the ENSAT or other multicentre studies. Clinical data were collected by reviewing medical records and re-evaluating clinical data for each patient during this period. The registry includes the following data for each patient: sex, age at the time of diagnosis, body weight and body mass index (BMI) at diagnosis, hormonal activity verified by laboratory results, Weiss-score, Ki67-index, primary tumour size, ENSAT stage and resection status (R0=complete resection, R1=microscopic residual tumour, R2=macroscopic residual tumour, Rx=cannot be determined) after adrenalectomy. The tumour stage was determined using the ENSAT criteria for all patients (13).

For the calculation of the S-GRAS prognostic score, we adhered to the criteria outlined in the original publication introducing this model (18): ENSAT stage (0 point for stages 1 and 2, 1 point for stage 3, and 2 points for stage 4), grade (0 point for ≤ 9%, 1 point for 10-19% and 2 points for ≥ 20% Ki67-index), resection status (0 point for R0, 1 point for Rx, 2 points for R1 and 3 points for R2), age (0 point for <50 and 1 point for ≥ 50 years), symptoms (1 point for hormonal symptoms at presentation). Patients deemed unresectable at the time of histological diagnosis were classified as R2 at scoring, reflecting the poor prognostic impact of the residual tumour mass. For the validation sub-analysis, we created four S-GRAS subgroups (0-1, 2-3, 4–5 and 6–9 points) that align with the divisions used in the original publication (18).

For the calculation of inflammation-based scores, a complete blood count obtained within 30 days prior to adrenalectomy or tumour biopsy was used for each patient, and the calculations were performed as follows: NLR by dividing the absolute neutrophil count by the absolute lymphocyte count and PLR by dividing the absolute platelet count by the absolute lymphocyte count. All patients with coexisting inflammatory diseases, concomitant infections, known autoimmune and/or haematological diseases were excluded from further analysis. To determine whether any of these conditions were present at the time of sample collection, the complete medical documentation of the relevant hospital admission was reviewed for each patient. To determine the cut-off values for NLR and PLR, we applied the thresholds of 5 and 190 respectively, which have been commonly used in a variety of solid tumours (29) and have previously proven to be appropriate in ACC patient cohorts as well (24, 30).

The frequency of follow-up visits and control CT scans was determined according to the actual guideline recommendations (31–33). For each patient, follow-up time was calculated from the date of histological diagnosis to the last visit to our Institution. OS was defined as the interval from the histologically confirmed diagnosis to death or last recorded contact. DFS was calculated for all patients who underwent R0 adrenalectomy and was defined as the interval between adrenalectomy and the first radiological detection of either a local recurrence or a distant metastasis. Patients who were not confirmed to be alive by the end of the follow-up period and for whom we did not have information regarding the date of their death were considered lost to follow-up. Survival rates at 24 and 60 months were calculated to assess the influence of NLR, PLR and each clinicopathological factor on OS.

### 2.2 Statistical analysis

We presented categorical variables as frequencies and percentages. All continuous variables were tested for Gaussian distribution with the Kolmogorov-Smirnov test. Normally distributed data were presented as means and standard deviations, whilst not normally distributed data were presented as medians and ranges (minimum-maximum). The distributions of continuous variables were compared using Mann-Whitney U test and were visualised with box-whiskers plots. Survival curves were generated using the Kaplan-Meier method, and their comparison was performed with log-rank tests, accompanied by pairwise comparisons, when appropriate. Univariate Cox proportional hazards models were employed to assess the association of each variable with overall survival. Specific variables demonstrating significant prognostic performance in the univariate analysis were subsequently entered into a multivariate Cox model. Hazard ratios (HR) with 95% confidence intervals (CI) were reported. Discriminative ability was quantified using Harrell’s concordance index (C-index), with 95% CIs derived from bias-corrected and accelerated (BCa) bootstrap resampling (1,000 iterations) due to our moderate sized cohort and limited number of events. A C-index of 0.5 was considered to correspond to no discrimination, 0.6-0.7 to acceptable discrimination, 0.7-0.8 to good discrimination and above 0.8 to excellent discrimination. Interaction between the S-GRAS score and NLR was explored by including mean-centred variables and their interaction term in a multivariate Cox regression model. The independent and complementary prognostic contribution of these scores was assessed using likelihood ratio test comparing nested Cox models. The significance level was set at a p-value lower than 0.05 for all analyses. Calculations were performed either using the IBM SPSS Statistics for Windows, version 27.0. Armonk, NY: IBM Corp or in R version 4.5.3; Foundation for Statistical Computing, Vienna, Austria.

## 3 Results

### 3.1 Patient cohort

Ninety-two patients with histologically confirmed adrenocortical carcinoma were treated between 2000 and 2023 at our Institution. Our retrospective study identified 67 patients with sufficient data to calculate the S-GRAS score. ENSAT stage II, a Ki67-index above 20%, R0 resection status and hormonal activity were the most frequent features in our patient cohort. 60 out of 67 patients underwent adrenal resection. Fifty-four patients (80.1%) received additional treatments (metastasectomy, mitotane, EDP-M chemotherapy, irradiation, etc.). The investigated clinicopathological factors are presented in **Table 1**.

**Table 1:** Clinicopathological features of ACC patients (N = 67).

Age (median, min-max)	59.9 (19.0-84.2)
<50 years at the time of diagnosis (n; %)	36 (53.7%)
≥50 years at the time of diagnosis (n; %)	31 (46.3%)
Gender (n; %)	
Male	24 (35.8%)
Female	43 (64.2%)
Hormonal activity (n; %)	
Active	42 (62.7%)
Cortisol	15 (22.4%)
Androgens (Testosterone, DHEAS)	4 (6.0%)
Aldosterone	2 (3.0%)
Multiple hormone excess	21 (31.3%)
Inactive	25 (37.3%)
Stage (n; %)	
ENSAT I	3 (4.5%)
ENSAT II	29 (43.3%)
ENSAT III	17 (25.4%)
ENSAT IV	18 (26.9%)
Ki67 index (n; %)	
0-9	17 (25.4%)
10-19	20 (29.9%)
≥20	30 (44.8%)
Resection state (n; %)	
R0	35 (52.2%)
R1	14 (20.9%)
R2	3 (4.5%)
Rx	8 (11.9%)

Our patient cohort had a median OS of 25 months (0–206). Survival at 24 months was 53.7%, and 16.4% at 60 months. Seven patients (10.4%) were lost to follow-up, whilst 19 (28.4%) were still alive at the end of the follow-up period. The median DFS for patients with an R0 resection status was 15 months (**Table 2**).

**Table 2:** Survival analysis.

Overall survival (months, median; min-max)	25 (0-206)
Lost to follow-up (n; %)	7 (10.4%)
Alive at the end of follow-up (n; %)	19 (28.4%)
Survival at 24 months (%)	53.7%
Survival at 60 months (%)	16.4%
Disease progression in patients with R0 resection state (n=30)	
Locoregional recurrence (n; %)	6 (18.6%)
Median time to locoregional recurrence (months, median; min-max)	17 (2-26)
Distant metastasis (n, %)	16 (50.0%)
Median time to distant metastasis (months, median; min-max)	18.5 (1-37)
Disease-free survival (months, median; min-max)	15 (1-37)

### 3.2 External validation of the S-GRAS scoring system

We calculated the S-GRAS scores for each patient, resulting in the following distribution across the S-GRAS subgroups: 8 (12.0%) patients had low (0–1) S-GRAS points, 23 (34.3%) patients had low-intermediate (2–3) S-GRAS points, 14 (20.9%) patients had high-intermediate (4–5) S-GRAS points and 22 (32.8%) patients had high (6–9) S-GRAS points. The subgroups were created by mirroring the distribution used in previous studies to compare survival results adequately (18–20).

Data regarding the relationship between each clinicopathological parameter and S-GRAS subgroups are presented in **Table 3**. Survival rates at 24 and 60 months are shown as percentages. When evaluating the components of the prognostic model separately, we found that ENSAT stage IV (p=0.014), a Ki67-index above 20% (p=0.009), resection statuses R1 (p=0.003) and Rx (p=0.014) and hormonal activity (p=0.032) were associated with a significantly increased risk of mortality. Our calculations indicated that higher S-GRAS scores correlated with progressively greater hazard ratios of mortality: HR = 8.9 (p=0.005) for patients with 4–5 points and HR = 16.2 (p<0.001) for patients with 6–9 points. Notably, the discriminative ability of the S-GRAS score (C-index= 0.765) exceeded that of its individual constituents, validating the added value of the scoring system.

**Table 3:** Validation of the S-GRAS prognostic scoring system (n=67).

	No of patients	Survival at 24 months (%)	Survival at 60 months (%)	Overall survival (months; min-max)	HR of mortality (95% CI)	p-value (HR)	C index	95% CI (BCa bootstrap)
ENSAT							0.702	0.639 – 0.702
I.	3	100	33	36 (35-73)	1.0			
II.	29	69	24	38 (9-206)	1.8 (0.2-13.9)	0.559		
III.	17	53	18	24 (0-104)	2.8 (0.4-21.8)	0.324		
IV.	18	22	0	14 (1-26)	**13.3 (1.7-106.3)**	**0.014**		
Ki67 index							0.638	0.547 – 0.711
0-9	17	76	24	36 (3-206)	1.0			
10-19	20	55	20	28 (8-104)	1.9 (0.8-4.5)	0.144		
≥20	30	40	10	17 (0-124)	**2.9 (1.3-6.5)**	**0.009**		
Resection status							0.675	0.58 – 0.758
R0	35	66	26	38 (2-206)	1.0			
R1	14	57	7	25 (0-104)	3.1 (1.5-6.4)	0.003		
R2	3	67	0	25 (8-42)	2.3 (0.5-10.1)	0.266		
Rx	8	25	13	14 (9-94)	**3.1 (1.2-7.5)**	**0.014**		
Age							0.54	0.471 – 0.61
<50 years	36	61	19	30 (1-206)	1.0			
≥50 years	31	45	13	20 (0-124)	1.2 (0.7-2.2)	0.454		
Hormonal activity							0.592	0.518 – 0.661
No	25	68	24	32 (1-206)	1.0			
Yes	42	46	12	22 (0-104)	**2.0 (1.1-3.8)**	**0.032**		
S-GRAS group							0.765	0.692 - 0.821
0-1p	8	88	63	70 (14-206)	1.0			
2-3p	23	70	17	34 (9-124)	4.2 (0.9-18.7)	0.058		
4-5p	14	50	7	24.5 (9-94)	**8.9 (1.9-40.7)**	**0.005**		
6-9p	22	27	5	14 (0-104)	**16.2 (3.7-71.8)**	**<0.001**		

Survival at 24 and 60 months, overall survival, hazard ratios of univariate Cox regression analyses and Harrell’s concordance-indexes calculated for each S-GRAS constituent and subgroups.

CI, confidence interval; HR, hazard ratio; C-index, Harrell’s concordance index.

All variables treated as categorical.

95% confidence intervals estimated by bias-corrected and accelerated (BCa) bootstrap resampling (1,000 iterations). A C-index of 0.5 was considered to correspond to no discrimination, 0.6-0.7 to acceptable discrimination, 0.7-0.8 to good discrimination and above 0.8 to excellent discrimination.

Data presented in bold correspond to statistically significant results.

**Figure 1:**
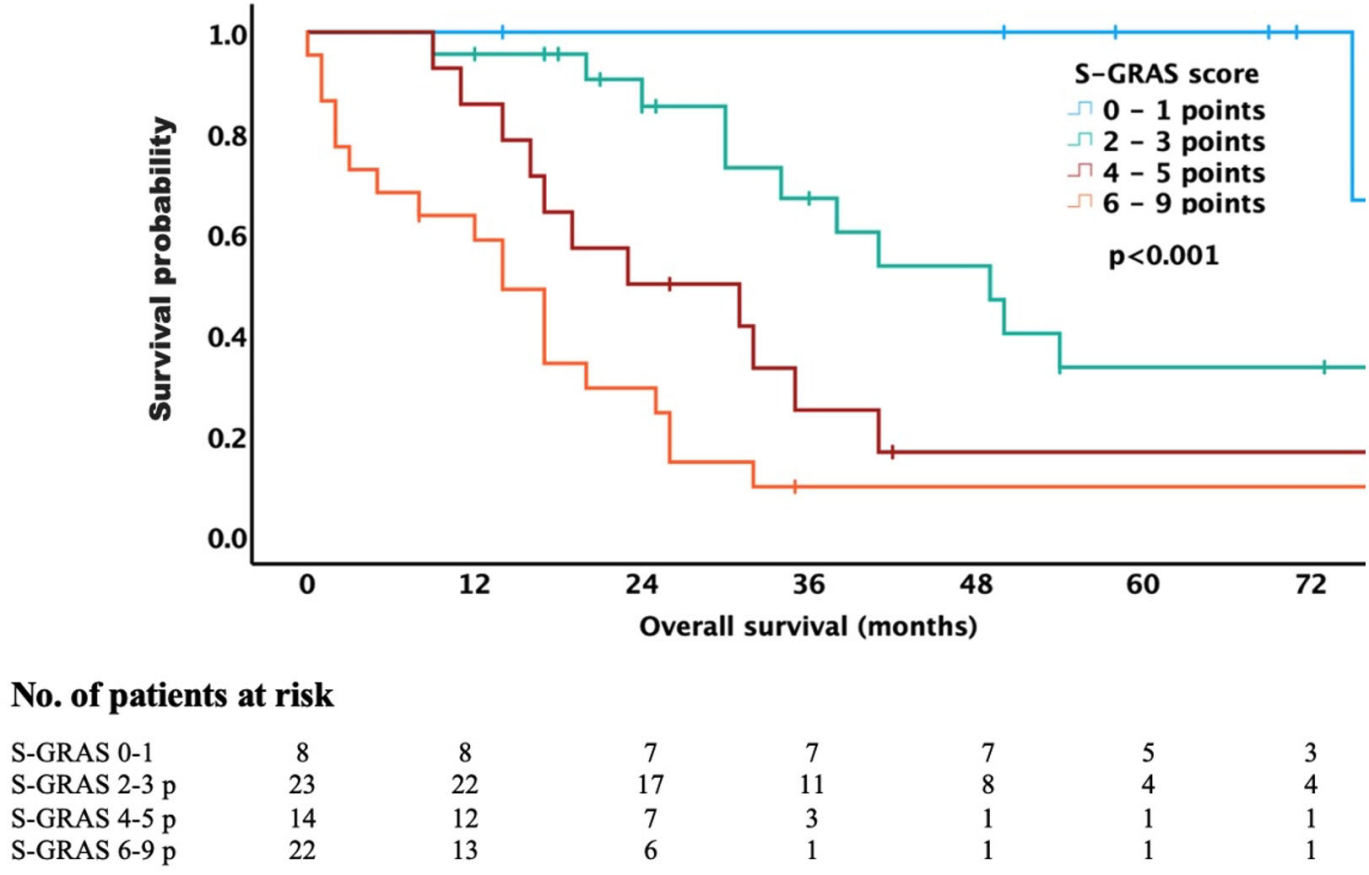
Kaplan-Meier curves displaying overall survival according to S-GRAS score groups. Censored patients are indicated by vertical tick marks on the survival curve.

The Kaplan-Meier curves illustrating OS according to S-GRAS subgroups are presented in **Figure 1** (p<0.001). Pairwise comparisons revealed significant differences between all subgroups (p=0.014 between the 0-1- and 2-3-point subgroups and p=0.035 between the 2-3- and 4-5-point subgroups), with the exception of the comparison between patients with 4–5 and 6–9 S-GRAS points (p=0.12).

### 3.3 Prognostic performance of inflammation-based scores

Among the 67 patients with available S-GRAS scores, a complete blood count for the calculation of inflammation-based scores was available for 46 cases. As four patients met one or more exclusion criteria, NLR and PLR analyses were ultimately conducted on a cohort of 42 patients. All subsequent calculations were performed on this patient cohort of 42. The mean NLR was 4,38 (± 2,94), and the mean PLR was 187,66 (± 83,31).

To assess the individual prognostic influence of these ratios on overall survival, we applied the cut-off values of 5 for NLR and 190 for PRL, both externally established in ACC survival analyses (29, 30). 29 (69.0%) patients had a NLR below this threshold, whilst 13 (31.0%) patients had a ratio above it. Regarding PLR, 24 (57.1%) patients had values below 190, whereas 18 (42.9%) patients had values exceeding this cut-off.

**Figure 2:**
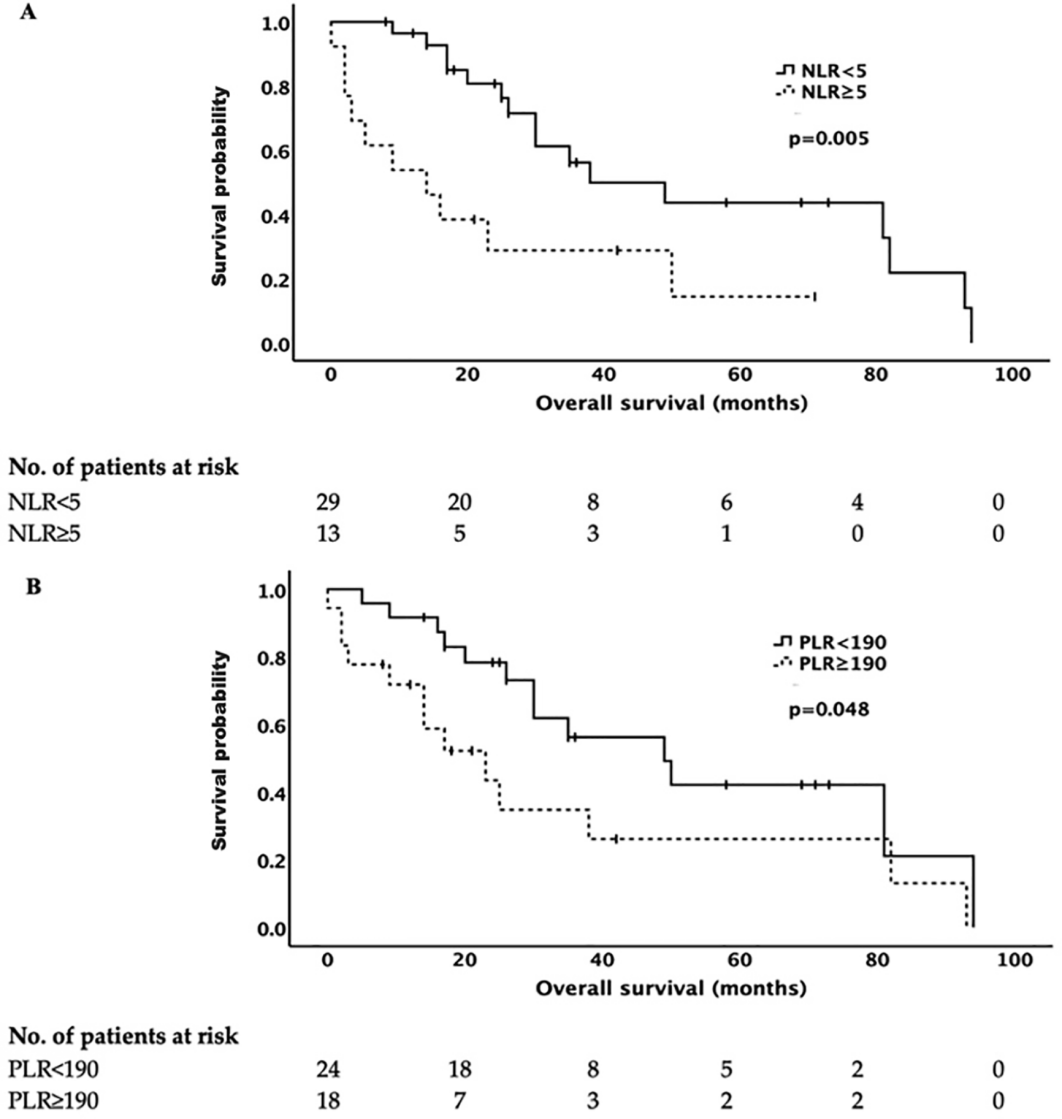
Kaplan–Meier survival curves showing overall survival in relation to **(A)** the NLR (neutrophil-to-lymphocyte ratio) and **(B)** the PLR (platelet-to-lymphocyte ratio). Censored observations are indicated by vertical tick marks on the survival curves.

Of the two investigated inflammation-based scores, patients with NLR≥5 were found to have a significantly increased hazard ratio for mortality (HR = 3.1; p=0.008). In contrast, the corresponding analysis for PLR, with a cut-off value of 190, yielded a marginally non-significant result. Kaplan–Meier curves illustrating overall survival based on the inflammation-based scores are presented in **Figure 2**, along with the results of the comparative log-rank tests for both NLR (p=0.005) and PLR (p=0.048).

### 3.4 Interaction between the S-GRAS score and NLR

**Figure 3:**
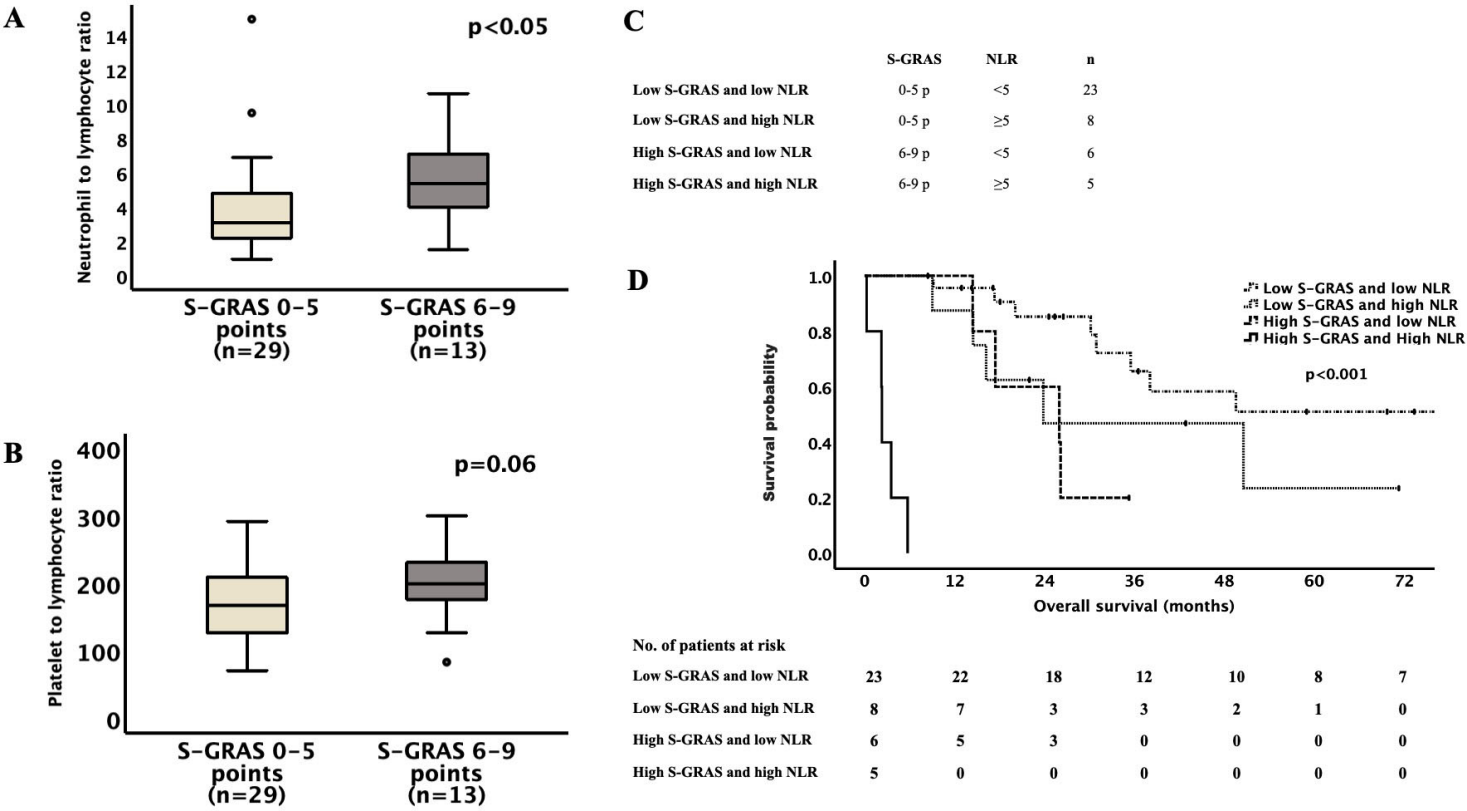
Interaction between the S-GRAS scoring system and NLR. The distributions of **(A)** NLR and **(B)** PLR were analysed in relation to S-GRAS scores. **(C)** Four subgroups (low S-GRAS and low NLR, low S-GRAS and high NLR, high S-GRAS and low NLR, high S-GRAS and high NLR) were created to visualize the interaction between the two metrics on a **(D)** Kaplan-Meier curve.

To assess whether these prognostic scores can be considered independent, we aggregated the previously described four S-GRAS subgroups into a low-intermediate (0–5 points) and a high (6–9 points) category. NLR was significantly higher in patients with S-GRAS scores of 6–9 as compared to those with 0-5 (p=0.024, **Figure 3A**), whilst PLR showed a marginally non-significant trend upon comparison (p=0.06; **Figure 3B**). Since PLR failed to reach a significance both in the univariate hazard analyses and the between-group comparison, it was excluded from further analyses and all subsequent calculations were performed using NLR.

As NLR exhibited a non-random distribution across the two S-GRAS categories, we assumed a potential interaction between the two variables. To formally assess this, we constructed a multivariable Cox model incorporating mean-centred S-GRAS (S-GRASc) and NLR (NLRc) scores alongside their interaction (S-GRASc x NLRc). All variables were treated as continuous for this sub-analysis. All three terms reached significance in the model (S-GRASc HR = 1.652 and p<0.001; NLRc HR = 1.291 and p<0.001 and S-GRASc x NLRc HR = 1.177 and p=0.009).

These results suggested a synergistic relationship, in which the prognostic impact of NLR is amplified in patients with higher S-GRAS score. To illustrate this interaction, patients were stratified into four groups based on the previously used S-GRAS (0–5 points vs 6–9 points) and NLR (<5 vs. ≥5) thresholds (**Figure 3C**). Kaplan-Meier curves for these groups are presented on **Figure 3D**. Patients with low S-GRAS and low NLR demonstrated the most favourable survival, whilst those with elevated values for both markers exhibited strikingly high mortality. The two intermediate groups (low S-GRAS/high NLR and high S-GRAS/low NLR) exhibited crossing survival curves.

### 3.5 Added prognostic value of NLR beyond S-GRAS

Having identified a significant interaction between S-GRAS and NLR we aimed to determine whether their combination offers superior discrimination compared to their individual utilisation. We constructed three individual Cox proportional hazard models (**Table 4**). In the univariate analysis, each unit increase of S-GRAS (model 1) was associated with a 53% increase in the hazard mortality (p<0.001), with excellent discriminative ability (C-index=0.803). NLR alone (model 2) also demonstrated a significant prognostic value, with each unit increase corresponding to a 19% elevation in mortality risk (p=0.002) and good discrimination (C-index=0.722).

**Table 4:** Univariate and multivariate Cox regression of S-GRAS and NLR models with discrimination analysis for overall survival.

	Cox-regression model	HR of mortality (95% CI)	p-value	Harrell’s C-index	95% CI (BCa bootstrap)	Likelihood ratio test
Model 1: S-GRAS alone	univariate	1.531 (1.232 – 1.902)	<0.001	0.803	0.704 – 0.874	
Model 2: NLR alone	univariate	1.193 (1.069 – 1.332)	0.002	0.722	0.583 – 0.834	
Model 3: S-GRAS and NLR	multivariate	S-GRAS: 1.581 (1.25 – 2.001)	**<0.001**	**0.822**	**0.728 – 0.903**	**vs. Model 1: χ²=7.72, p=0.006**
		NLR: 1.207 (1.075 – 1.356)	**<0.001**			**vs. Model 2: χ²=15.46, p<0.001**

HR, hazard ratio; CI, confidence interval; C-index, Harrell’s concordance index.

All variables treated as continuous.

95% confidence intervals estimated by bias-corrected and accelerated (BCa) bootstrap resampling (1,000 iterations). A C-index of 0.5 was considered to correspond to no discrimination, 0.6-0.7 to acceptable discrimination, 0.7-0.8 to good discrimination and above 0.8 to excellent discrimination.

Data presented in bold correspond to the demonstration of the superior prognostic value of our combined model.

After combining the predictors in the multivariate model, both S-GRAS (HR = 1.581, p<0.001) and NLR (HR = 1.207, p<0.001) retained their significance. Importantly, the combined model (model 3) achieved an excellent discrimination (C-index=0.822) that exceeded what each variable provided alone. Likelihood ratio tests formally confirmed the independent contribution of each prognostic tool: adding NLR to model already containing S-GRAS (model 1 vs. model 3) or S-GRAS to a model already containing NLR (model 2 vs. model 3) produced a significant improvement in model fit (χ²=7.72, p=0.006 and χ²=15.46, p<0.001, respectively).

## 4 Discussion

In this study, we evaluated two increasingly recognised prognostic tools of ACC and characterised their interrelationship. While the well-established S-GRAS score provided modestly superior prognostic performance compared to the single parameter of NLR, their combined application demonstrated significantly increased predictive value that exceeded both measures alone. In addition, we identified a synergistic interaction between S-GRAS and NLR, most pronounced in high-risk patients with concurrently increased S-GRAS score and NLR values. In addition to our novel findings, we performed an external validation of the S-GRAS model on what is, to the best of our knowledge, the largest ACC cohort originating form a single-centre.

Managing rare diseases without robust recommendations supported by high-level evidence presents a significant challenge. Thus, single-centre experiences confirming results from larger patient cohorts may enhance their value over time. Among the components of the S-GRAS score, we found that a higher ENSAT stage, a Ki67-index above 20%, R1 and Rx resection statuses and hormonal activity were associated with increased risk of mortality. The lack of significant results for the R2 status is likely due to its low incidence rate. By combining these clinicopathological factors into a single numeric parameter, the S-GRAS score, we could correlate higher scores with an increased risk of death. The S-GRAS score proved to be a more sensitive prognostic tool that provides better discrimination than its separate components. Our survival analysis comparison indicates that our patients’ survival is not different from those of larger multi-centre studies despite the less robust performance of statistical tests (18–20). The outcomes of our retrospective, institutional analysis demonstrate the usability and reliability of the S-GRAS scoring system, as this model has proven to be a statistically significant prognostic tool even when applied to a smaller number of cases, following the validation of several multi-centre (17–20, 34), and a small single-centre study (21) (**Supplementary Table 1**).

Several modified or updated versions of the S-GRAS scoring system have been proposed in recent years. For paediatric ACC patients, the pS-GRAS system was developed (35), and subsequently validated by a multicentric study, establishing the model as a new prognostication tool (36). No robust comparative data is available on whether the pS-GRAS score or the previously proposed Five-Item score and Children’s Oncology Group’s (COG) staging criteria exhibit superior prognostic value (37). The use of molecular biological markers, alongside standard clinicopathological factors, has gained recognition in recent years for achieving even more accurate prognostication in patients. When combining tumour stage and proliferation index with molecular classification, the prognostic value is significantly enhanced compared to using only molecular or clinical parameters in patients with localised ACC [30]. Recently, a new prognostic tool, the COMBI score, was proposed [31]. Integrating genetic, epigenetic and molecular markers into current clinical scoring systems results in the most accurate prognostication for ACC patients, potentially allowing for more individualised patient management. However, to reliably assess and compare these novel versions of the S-GRAS score, a standardised, uniform histopathological and molecular methodology should be developed, which is significantly hindered by the generally multicentric management of these patients. Since most molecular factors are not yet assessable and evaluated in an everyday clinical setting, the clinical S-GRAS system currently provides the best single prognostic score for routine patient care.

Inflammation-based scores, as opposed to the previously mentioned genetic markers, are easily accessible and cost-effective, making them widely applicable in clinical practice. These scores have been shown to predict tumour progression and clinical outcomes by reflecting systematic inflammation status (22). In particular, a substantial number of studies have reported associations between NLR (38), PLR (39) and patient survival outcomes. In studies involving ACC patient cohorts, higher values are associated with shorter overall survival, an increased risk of recurrence or distant metastasis, as well as more aggressive clinicopathological characteristics (24–27, 30). Similar associations have also been established for paediatric ACC patients (40). NLR and PLR have also been used to differentiate between benign and malignant adrenal tumours (41, 42), to predict response to first-line chemotherapy (43) or to differentiate between the types of endogenous hypercortisolism (44) and steroid patterns in adrenocortical tumours (42). Our findings support NLR as an independent predictor of survival. A similar assessment of PLR demonstrated a non-significant trend, which my become more apparent in larger cohorts. Nonetheless, NLR emerged as the more robust inflammation-based marker in our own analysis.

A key contribution of this study is the characterisation of the relationship between the S-GRAS score and NLR in the context of ACC prognosis. The extent of systematic inflammation is influenced not only by tumour status but by several additional factors as well. Among the components of the S-GRAS score, both older age and hormonal excess (42, 44) may contribute to an enhanced systemic inflammatory state. These factors can potentially explain why we were able to associate higher S-GRAS scores with higher NLR measures. This dependant distribution is what prompted us to conduct a formal interaction analysis. This demonstrated that whilst both factors independently predict mortality, their combined effect on survival is greater than what either variable predicts alone. These two findings – independence and synergy – are not contradictory: independence means each variable captures information the other lacks (confirmed by our subsequent likelihood ratio test), whilst synergy means that when both are elevated, the joint hazard exceeds what their simple addition would suggest. This synergistic interaction between the two measures is primarily attributable to patients with extreme values, as presented in our four-group Kaplan-Meier analysis. In contrast, the intermediate risk groups (characterised by discordant score profiles: low S-GRAS with high NLR or high S-GRAS with low NLR) exhibited repeatedly crossing survival curves. This pattern suggests an interaction of a different dynamic: when the two scores disagree, neither alone provides a stable prognostic signal but rather a time-dependent variability that requires careful, personalised clinical interpretation. However, we acknowledge the small cohort as a serious caveat of our analysis and suggest that similar calculations on more extensive cohorts might be able to better outline the interaction in patients with discordant prognostic scores.

Following the original submission of our paper, a recently published multicentric ENSAT study seems to converge with our findings (30). Mangone et al. constructed a novel clinicopathological scoring system for patients with advanced ACC and effectively used NLR to predict survival and treatment response in the subgroup. While NLR carries prognostic value across the full ACC spectrum, our results suggest that its additional contribution is greatest in patients identified as high-risk by the S-GRAS score – exactly the population the study has focused on.

Following the identification of an interaction between the two parameters, we next evaluated their clinical utility, specifically, whether their combined application improves prognostic performance compared to each marker alone. To this end, we constructed three simple models to predict mortality and observed that the model incorporating both S-GRAS and NLR achieves superior prognostic discrimination. Due to our limited sample size, we found it important to better characterise this increment in predictive value and confirmed with likelihood ratio tests that the difference between our models is indeed significant. By doing so, following the findings of several studies addressing S-GRAS (18–21) and NLR (24, 27) separately, our findings extend the existing evidence by describing that these metrics capture complementary predictive values. Therefore, utilising both is recommended as it provides enhanced prognostic stratification compared to either measure alone.

We acknowledge several limitations to our study. First, the retrospective nature of our analysis neither accounts for the heterogeneity of treatment modalities applied, nor the evolving trends in ACC patient management over the past two decades. Histological evaluation of tumour samples were not conducted using standardised methods throughout the entire follow-up period. Second, whilst results align with previously published findings on ACC prognosis, the relatively small sample size limited the scope of formal prognostic assessment. Some of the statistical approaches applied ideally require a larger patient cohort with a greater number of events. We aimed to account for this to the fullest with our methodology, but our results are to be interpreted cautiously. Third, as a single-centre cohort, our patient population does not reflect the broader spectrum of ACC patient characteristics and management approaches. We believe a key strength of our publication lies in the meticulous re-evaluation of medical records and the comprehensive follow-up of patients treated at our centre. Although we consider a loss to follow-up ratio of 10.4% excellent for a study reaching back 23 years, it could represent a potential bias when assessing OS. Another strength of our analysis is the methodological approach that was designed to mitigate the limitations imposed by our moderate cohort size. We believe that in addition to the previously noted limitations of our single-centre approach, it also holds additional value by adequately representing the patient management of a national referral centre.

In conclusion, using NLR in addition to the well-established S-GRAS score is recommended for ACC patients, as the predictive value of these independent markers complement each other. A significant synergistic interaction between the two metrics indicates that their joint prognostic impact exceeds what each predicts independently, with the greatest clinical utility to be exploited in high-risk patients, with elevated S-GRAS scores. This study also provides an external validation of the S-GRAS scoring system in an independent cohort.

## Data Availability

The original contributions presented in the study are included in the article/**Supplementary Material**. Further inquiries can be directed to the corresponding author.
