# Supplementary material for: S-GRAS score and the complementary prognostic value of neutrophil-to-lymphocyte ratio in adrenocortical carcinoma: evidence for a synergistic interaction
Source: Front Endocrinol (Lausanne). 2026 Jun 8;17:1733138. doi: 10.3389/fendo.2026.1733138 (PMC13283844; doi:10.3389/fendo.2026.1733138)
Supplement: Supplementary file 1 [file Table1.docx]

**Table S1.** Brief summary of publications analysing the prognostic value of S-GRAS scoring system.

| Author (year) | Single centre / Multicentre | Number of patients | ENSAT stages | Grade (Ki67-index) | Resection status | Age | Hormonal symptoms | No of patients by  S-GRAS groups | Score subgroups associated  with PFS, OS or DFS | Survival (months) | Cox regression (HR; 95% C.I.) | |
| --- | --- | --- | --- | --- | --- | --- | --- | --- | --- | --- | --- | --- |
| **Elhassan et al. (2022)** | Multicentre | 942 | I: 86  II: 474  III: 218  IV: 164 | Ki67<9%: 264  9%≤Ki67<19%: 216  Ki67≥20%: 462 | R0: 648  Rx: 104  R1: 56  R2: 134 | <50: 466  ≥50: 476 | No symptoms: 277  Symptoms: 665 | S-GRAS 0-1p: 168  S-GRAS 2-3p: 366  S-GRAS 4-5p: 225  S-GRAS 6-9p: 183 | ***Univariate Cox-regression analysis (PFS)*** | | | |
|  |  |  |  |  |  |  |  |  | S-GRAS 2-3p  S-GRAS 4-5p  S-GRAS 6-9p | 33.0  10.0  6.0 | 2.8 (2.0 – 4.0)  6.4 (4.5 – 9.0)  11.5 (8.1– 16.3) | |
| **Lippert et al. (2022)** | Multicentre | 237 | I: 14  II: 106  III. 71  IV:45  *NDA* *: 1 | Ki67<9%: 56  9%≤Ki67<19%: 57  Ki67≥20%: 113  *NDA**: 11 | R0: 169  Rx: 22  R1: 18  R2: 18  *NDA **: 10 | <50: 117  ≥50: 120 | No symptoms: 109  Symptoms: 128 | S-GRAS 0-1p: 37  S-GRAS 2-3p: 100  S-GRAS 4-5p: 67  S-GRAS 6-9p: 33 | ***Univariate Cox-regression analysis (OS)*** | | | |
|  |  |  |  |  |  |  |  |  | S-GRAS 4-5p  S-GRAS 6-9p | *NDA* * | 5.0 (2.4 – 10.3)  8.8 (4.0 – 19.1) | |
| **Mihai (2024)** | Single centre | 31 | I: 1  II: 14  III: 7  IV: 9 | *NDA.* * | R0: 23  R1: 7  R2: 1  Rx: 0 | *NDA* * | Inactive: 19  Active: 12 | S-GRAS 0-1p: 9  S-GRAS 2-3p: 6  S-GRAS 4-5p: 10  S-GRAS 6-9p: 6 | ***Univariate Cox-regression analysis (DFS)*** | | |  |
|  |  |  |  |  |  |  |  |  | S-GRAS 2-3p  S-GRAS 4-5p  S-GRAS 6-9p | 94.7  22.9  8.2 | 1.75  6.2  20.7 | |
| **Baek et al. (2024)** | Multicentre | 114 | I: 13  II: 54  III: 17  IV: 30 | Ki67<9%: 44  9%≤Ki67<19%: 33  Ki67≥20%: 37  *NDA.* *: 11 | R0: 70  R1: 7  R2: 30  Rx: 7 | <50: 53  ≥50: 61 | No symptoms: 60  Symptoms: 54 | S-GRAS 0-1p: 28  S-GRAS 2-3p: 43  S-GRAS 4-5p: 11  S-GRAS 6-9p: 32 | ***Univariate Cox-regression analysis (DFS)*** | | |  |
|  |  |  |  |  |  |  |  |  | S-GRAS 4-5p  S-GRAS 6-9p | *NDA.* * | 3.18 (1.2 – 8.5)  69.5 (26.0 – 185.7) | |
| **Bényei et al. (2025)** | Single centre | 67 | I: 3  II: 29  III: 17  IV: 18 | Ki67 <10%: 17 10%≤Ki67<20%: 20  Ki67 ≥20%: 30 | R0: 35  R1: 14  R2: 3  Rx: 8 | <50: 36  ≥50: 31 | Inactive: 25  Active: 42 | S-GRAS 0-1p: 8  S-GRAS 2-3p: 23  S-GRAS 4-5p: 14  S-GRAS 6-9p: 22 | ***Univariate Cox-regression analysis (OS)*** | | |  |
|  |  |  |  |  |  |  |  |  | S-GRAS 4-5p  S-GRAS 6-9p | 24.5  14.0 | 8.9 (1.9-40.7)  16.2 (3.7-71.8) | |
